# M2‐like macrophages polarized by Foxp3^−^ Treg‐of‐B cells ameliorate imiquimod‐induced psoriasis

**DOI:** 10.1111/jcmm.17748

**Published:** 2023-04-19

**Authors:** Jing‐Hui Huang, Yu‐Li Lin, Li‐Chieh Wang, Bor‐Luen Chiang

**Affiliations:** ^1^ Graduate Institute of Clinical Medicine, College of Medicine National Taiwan University Taipei Taiwan; ^2^ Department of Medical Research National Taiwan University Hospital Taipei Taiwan; ^3^ Department of Pediatrics National Taiwan University Hospital Taipei Taiwan; ^4^ Graduate Institute of Immunology, College of Medicine National Taiwan University Taipei Taiwan

**Keywords:** imiquimod, M2 macrophage, psoriasis, STAT6, Treg‐of‐B cells

## Abstract

Our group have demonstrated that splenic B cells contributed to the CD4^+^CD25^−^ naive T cells conversion into CD4^+^CD25^+^Foxp3^−^ regulatory T cells without adding appended cytokines, named Treg‐of‐B cells which were potent suppressors of adaptive immunity. We like to investigate whether Treg‐of‐B cells could promote alternatively activated macrophage (M2 macrophages) polarization and alleviate inflammatory disease, psoriasis. In this study, we co‐cultured the bone marrow‐derived macrophages (BMDMs) with Treg‐of‐B cells under LPS/IFN‐γ stimulation and analyzed the M2‐associated gene and protein using qPCR, western blotting, and immunofluorescence staining. We also examined the therapeutic effect of Treg‐of‐B cell‐induced M2 macrophage for skin inflammation using imiquimod (IMQ)‐induced psoriatic mouse model. Our results showed that BMDMs co‐cultured with Treg‐of‐B cells upregulated typical M2‐associated molecules, including Arg‐1, IL‐10, Pdcd1lg2, MGL‐1, IL‐4, YM1/2 and CD206. In an inflammatory environment, TNF‐α and IL‐6 production by macrophages co‐cultured with Treg‐of‐B cells was decreased significantly. The molecular mechanism revealed that Treg‐of‐B cells promoted M2 macrophage polarization via STAT6 activation in a cell contact‐dependent manner. Moreover, the treatment with Treg‐of‐B cell‐induced M2 macrophages attenuated the clinical manifestations of psoriasis, such as scaling, erythema and thickening in the IMQ‐induced psoriatic mouse model. T cell activation in draining lymph nodes was decreased in the Treg‐of‐B cell‐induced M2 macrophage group after IMQ application. In conclusion, our findings suggested that Foxp3^−^ Treg‐of‐B cells could induce alternatively activated M2 macrophages through STAT6 activation, providing a cell‐based therapeutic strategy for psoriasis.

## 1 INTRODUCTION

Macrophages play a crucial role in the dynamic nature of innate immunity. Evidence suggests that macrophages have phenotypic diversity and plasticity, and are influenced by environmental cues. Macrophages are activated by lipopolysaccharide (LPS) and T helper (Th) 1 cytokine interferon‐gamma (IFN‐γ) to promote M1 (classically activated) macrophage polarization, which has proinflammatory effects. M1 macrophages are characterized by the expression of Toll‐like receptor, inducible nitric oxide synthase, CD80 and CD86. In contrast, M2 (alternatively activated) macrophage polarization was originally observed in response to Th2 cytokines, which exert anti‐inflammatory, immunomodulatory and tissue repair effects.^1^ M2 macrophages are subdivided into M2a, M2b and M2c. Interleukin (IL)‐4 and IL‐13 activate M2a and M2b macrophages by immune complexes, and M2c macrophages are induced by transforming growth factor beta (TGF‐β), IL‐10 and glucocorticoids.^2^ M2 macrophages express low levels of inflammatory markers, such as tumour necrosis factor‐alpha (TNF‐α) and inducible nitric oxide (iNOS), and high levels of anti‐inflammatory factors, such as IL‐10, Arg‐1 and CD206.^3^ A previous study showed that monocytes co‐cultured with expanded regulatory T cells (Treg) had reduced major histocompatibility complex class II expression accompanied by upregulation of M2 markers, CD206 and IL‐10.^4^

Treg cells are important in maintaining immune tolerance and preventing autoimmunity.^5^ Naturally occurring Treg cells are broadly classified into thymus‐derived Treg (tTreg) cells and peripheral Treg cells (pTreg cells), which exert forkhead box p3 (Foxp3)‐dependent immunosuppressive effects.^6^ Treg cells suppress the activation and proliferation of immune cells via multiple mechanisms. For example, the release of TGF‐β and IL‐10 enables Treg cells to suppress the proliferative response of effector T cells.^7^ Moreover, Treg cells modulate antigen‐presenting cells (APCs) function via interfering its maturation via the interaction of lymphocyte‐activation gene 3 (LAG3)/major histocompatibility complex class II (MHC II) and cytotoxic T lymphocyte antigen‐4 (CTLA4) /CD80/CD86‐mediated induction of indoleamine 2, 3 dioxygenase (IDO).^7^ In contrast, Foxp3^−^ type 1 regulatory T cells do not express Foxp3, which regulates innate immunity.^8^ A previous study showed that type 1 regulatory T cells suppress macrophage inflammasome activation, resulting in reduced caspase‐1 activation and IL‐1β production.^8^

B cells have been found to play a critical role in maintaining and inducing immune tolerance. B cell‐deficient mice developed worsening and delayed recovery from EAE disease, suggesting the role of B cells in immune tolerance.^9^ Previous studies revealed that resting B cells could expand the Treg population via TGF‐β or in an allogenic condition.^10^, ^11^ In addition, thymus‐derived B cells played a critical role in the development of thymic Treg cell precursors and the proliferation of mature thymic Treg cells.^12^ A previous study indicated that splenic B cells induced regulatory T cells in the presence of a mature immunological synapse.^13^ Our group demonstrated that Peyer's patch B cells could convert naïve T cells into Foxp3^−^ Treg cells, referred to as Treg‐of‐B cells.^14^ After that, we also found that peritoneal B‐1a cells and splenic B2 cells could generate functional CD4^+^Foxp3^−^ Treg cells, which have similar Treg‐associated characteristics, such as the expression of PD‐1, LAG3, ICOS, GITR, CTLA4 and OX40, and produced IL‐10.^15^, ^16^, ^17^ With regard to in vivo application, Treg‐of‐B cells could suppress Th2‐mediated allergic asthma, joint inflammation in collagen‐induced arthritis and Th1/Th17‐mediated intestinal inflammation and gouty inflammation.^14^, ^16^, ^17^, ^18^, ^19^ In addition, other study indicated that adoptive transfer of B cells into the B cell‐deficient mice (μMT mice) contributed to the restoration of Treg cells number in these mice and prevent DSS‐induced colitis.^20^ Furthermore, we reported that Treg‐of‐B cells could modulate innate immunity via inhibiting LPS/ATP and monosodium urate (MSU)‐induced NOD‐, LRR‐ and pyrin domain‐containing protein 3 (NLRP3) inflammasome activation in macrophages.^19^

Psoriasis is a chronic inflammatory skin disorder characterized by erythema, scaling, thickening and systemic inflammation and it affects 2–4% of the general population.^21^ Although the pathogenic mechanisms are still unclear, recent studies have indicated that macrophages play a crucial role in psoriasis‐like skin inflammation. A previous study showed that patients with severe psoriasis have a high ratio of M1/M2 macrophages.^22^ Epithelium/dermis‐lining macrophages have been observed in human psoriasis.^23^ Other studies have also indicated that activated macrophage recruitment to skin lesions is critical for the maintenance of psoriasis.^24^ M1 macrophages can activate Th1/Th17 cells and suppress M2 macrophages by producing IFN‐γ, IL‐23 and IL‐17.^25^ In psoriasis, dysregulation of Th1 and Th17 infiltration into the dermis of inflamed skin lesions contributes to activation of the IL‐23/IL‐17 axis, which stimulates keratinocytes to amplify the inflammatory response.^26^ Therefore, targeting M1 macrophage polarization is a potential therapeutic strategy for the treatment of psoriasis.

A previous study indicated that human CD4^+^ CD25^+^ FoxP3^+^ Tregs induce alternative CD163^+^/CD206^+^ human monocytes/macrophages in both a cell contact‐ and soluble factor‐dependent manner.^27^ We indicated that Treg‐of‐B cells suppressed LPS/ATP and MSU‐induced NLRP3 inflammasome activation in macrophages by inhibiting nuclear factor‐kappa B (NF‐κB) signalling.^19^ It has been demonstrated that the induction of NLRP3‐associated protein and mRNA expression is inhibited in M2 macrophages.^28^, ^29^ We investigated whether Treg‐of‐B cells could induce M2 macrophage polarization, and studied the molecular mechanism underlying Treg‐of‐B‐induced M2 macrophages. Moreover, we used topical application of the TLR7/8 ligand, imiquimod (IMQ), to induce psoriasis‐like skin inflammation in a mouse model. We also further explored the therapeutic potential of M2 macrophages in an animal model of psoriasis and provided a novel cell‐based therapeutic approach for inflammation disease.

## 2 MATERIALS AND METHODS

### 2.1 Animals

Six‐to‐eight weeks old male BALB/c mice were purchased from the National Animal Center. STAT6^−/−^ mice were obtained from the Jackson Laboratory. All mice were maintained at the Laboratory Animal Center of the College of Medicine, National Taiwan University. Animal experiments were approved by the Institutional Animal Care and Use Committee of the College of Medicine, National Taiwan University and were performed in accordance with the approved guidelines.

### 2.2 Induction of Treg‐of‐B cells

Splenic CD4^+^ CD25^−^ T cells and B220^+^ B cells were isolated from BALB/c mice. Splenic B220^+^ B cells were immunomagnetically purified using a BD IMag Cell Separation Magnet. Splenic CD4^+^ CD25^−^ T cells were purified by negative immunomagnetic selection using the EasySep Mouse CD4^+^ T Cell Isolation Kit (STEMCELL Technologies). CD4^+^CD25^+^ tTreg cells were obtained by incubating PE‐anti‐CD25 (BioLegend) and anti‐PE beads (BD Biolegend). Purified B cells were co‐cultured with purified splenic CD4^+^ CD25^−^ T cells under anti‐CD3/CD28 (0.5 μg/ml) stimulation for 3 days. Treg‐of‐B cells were obtained by depleting B220^+^ B cells.

### 2.3 Differentiation of bone marrow‐derived macrophages (BMDMs)

Bone marrow cells were harvested from the femurs and tibias of BALB/c and STAT6^−/−^ mice and cultured in 10% fetal bovine serum‐ RMPI medium supplemented with 15 ng/mL macrophage colony‐stimulating factor (MCSF, Peprotech) for 6 days to differentiate into macrophages.

### 2.4 In vitro co‐culture system

Wild‐type (WT) or STAT6^−/−^ BMDMs were co‐cultured with Treg‐of‐B cells at a 3:1 ratio overnight under anti‐CD3 and anti‐CD28 Abs (1 μg/mL) stimulation. The cultures were stimulated with LPS (100 ng/mL), IFN‐γ (50 ng/mL), or IL‐4 (50 ng/mL) for 24 h. The supernatant was collected and analyzed by ELISA. After removing the Treg‐of‐B cells, the BMDMs were lysed and the mRNA and protein expression were analyzed.

In a Transwell system, BMDMs were seeded in the bottom chambers, and Treg‐of‐B cells were cultured on the Transwell insert with 0.4 μM pore size under anti‐CD3/CD28 stimulation.

### 2.5 IMQ model and adoptive transfer of Treg‐of‐B cell‐induced M2 macrophages

Mice were treated with 62.5 mg of 5% IMQ cream (Aldara; 3M Pharmaceuticals) on the shaved dorsal skin daily for 4 consecutive days. On day 5, the mice were sacrificed, and the skin tissue, axillary lymph nodes (ALNs), inguinal lymph nodes (ILNs) and spleen were excised. Cells were harvested to examine local and systemic immune responses. Mice were randomly assigned to negative control (Vaseline), IMQ, Treg‐of‐B cell‐induced M2 macrophage and dexamethasone groups. For the treatment experiments, 5 × 10^5^ Treg‐of‐B cell‐induced M2 macrophages were adoptively transferred into mice via intravenous injection on days 0, 1 and 3. In the dexamethasone group, mice were administered 3 mg/kg dexamethasone daily via intraperitoneal injection.

### 2.6 Haematoxylin and eosin staining and scoring severity

Dorsal skin specimens were fixed with 10% formalin solution prior to paraffin embedding. Sections were stained with hematoxylin and eosin to examine immune cell infiltration into subcutaneous tissues and epidermal thickness. Epidermal thickness in the skin section was further determined using H and E staining.

To score the severity of skin inflammation in mice with psoriasis, the scoring system was based on the clinical Psoriasis Area and Severity Index. Scaling and erythema of the dorsal skin were scored on a scale of 0 (none) to 4 (very marked), as previously described.^30^ Scoring was performed in a double‐blind manner.

### 2.7 Western blotting of Arg‐1, p‐STAT6 and total STAT6

Western blotting was performed, as previously described.^31^ Cells were lysed with Triton X‐100‐based lysis buffer supplemented with phosphatase and protease inhibitors. Protein lysates were incubated at 95°C for 10 min. Samples were subjected to 10–15% sodium dodecyl sulfate‐polyacrylamide gel electrophoresis. Proteins were transferred to polyvinylidene fluoride membranes (PVDF, millipore) and incubated with antibodies against Arg‐1(Cell signalling #93668), p‐STAT6 (BD #558241), total STAT6 (BD, #612290) and β‐actin (Merck Millipore, AB_2223041). The ratio of phosphorylated STAT6 to the total basal STAT6 protein was quantified by performing densitometry on the immunoblots and analyzed using Image J.

### 2.8 Immunofluorescence staining

For in vitro experiment, macrophages were co‐cultured with or without Treg‐of‐B cells overnight. The co‐cultures were then stimulated with LPS (100 ng/mL) and IFN‐γ (50 ng/mL) for 24 h. After removing the Treg‐of‐B cells, the BMDMs were then stained with DAPI, Alexa 647‐F4/80, PE‐PDL2 and PE‐MGL‐1 to visualize their expression.

For in vivo experiment, skin sections from the IMQ mouse model were collected and stained with DAPI, Alexa 647‐F4/80 or Alexa 594‐TCR β to examine the infiltration of macrophages and T cells in the skin tissues.

### 2.9 Reverse‐transcription polymerase chain reaction

Total RNA from BMDMs, CD4^+^CD25^−^, CD4^+^CD25^+^ tTreg, Treg‐of‐B cells and skin tissue was extracted using TRIzol reagent. The extracted RNA (3 μg) was transcribed into complementary DNA using MMLV reverse transcriptase (Applied Biosystems). Gene expression was quantified using SYBR Green and the ABI 7500 Fast Real‐Time PCR System. The expression of *Arg1*, *Il‐10*, *Pdcd1lg2* (*PDL2*), *Mgl1*, *Il4*, *Ym1/2*, *Cd206*, *Tnfα*, *Il6*, *Il1β*, *Nos2*, *Cd86*, *Cd80*, *Il17f* and *Il23* was determined by normalization to *Gapdh* and analyzed by using the ΔΔCt method. The relative gene expression was determined by calculating 2^−ΔΔCt^. Primer sequences are listed in Table 1.

**TABLE 1:** The sequences of primers used for Q‐PCR.

Gene name	Forward 5′ → 3′	Reverse 5′ → 3′
Interleukin‐1β	agttgacggaccccaaaag	agctggatgctctcatcagg
Nos2	tgcatggaccagtataaggcaagc	gcttctggtcgatgtcatgagcaa
Tumour necrosis factor‐𝛂	tcagccgatttgctatctcat	agtacttgggcagattgacct
Gapdh	gatgggtgtgaaccacg	agatccacgacggacac
CD80	ggcaaggcagcaatacctta	ctctttgtgctgctgattcg
CD86	tctccacggaaacagcatct	cttacggaagcacccatgat
Pdcd1lg2	acgtggccacttcatgtttt	tcttgagggtttcccatcag
MGL‐1	tgagaaaggctttaagaactggg	gaccacctgtagtgatgtggg
Arg‐1	ccagaagaatggaagagtcagtgt	gcagatatgcagggagtcacc
IL‐23	ggtggctcagggaaatgt	gacagagcaggcaggtacag
IL‐10	tcggaaatgatccagttttac	tcactcttcacctgctccac
IL‐4	tgtcatcctgctcttctttctc	tctgtggtgttcttcgttgc
IL‐17F	catacccaggaagacatacttagaag	agtcccaacatcaacagtagc
CD206	gttcacctggagtgatggttctc	aggacatgccagggtcaccttt
YM1/2	agaagggagtttcaaacctggt	gtcttgctcatgtgtgtaagtga
IL‐6	agacaaagccagagtccttcag	tgccgagtagatctcaaagtga

### 2.10 Flow cytometry

To characterize the surface markers, the isolated cells were stained with fluorescence‐conjugated antibodies, which are listed in Table 2. Data acquisition and analysis were performed using the BD FACSLyric system (BD Biosciences) and FlowJo software (BD Biosciences).

**TABLE 2:** Fluorescence‐conjugated antibodies were used.

Ab name	Clone	Supplier
PE‐PD1	Clone J43	BD
PE‐OX40	Clone OX‐86	Biolegend
PE‐GITR	Clone DTA‐1	Biolegend
PE‐ICOS	Clone 15F9	Biolegend
PE‐LAG3	Clone C9B7W	BD
PE‐CD25	Clone 7D4	Biolegend
FITC‐CD25	Clone PC61	Biolegend
APC‐CD25	Clone PC61	BD
PerCP‐CD4	Clone RM4‐5	BD
PE‐Foxp3	Clone MF‐14	Biolegend
Alexa 647‐F4/80	Clone BM8	Biolegend
Alexa 594‐TCR	Clone H57‐597	Biolegend
PE‐PDL2	Clone TY25	Biolegend
PE‐MGL1	Clone LOM‐8.7	Biolegend

### 2.11 Determination of cytokine levels

To measure cytokine levels, Treg‐of‐B cells, CD4^+^ CD25^+^ Treg cells and CD4^+^ CD25^−^ T cells were restimulated with anti‐CD3/CD28 antibodies (1 μg/ml) for 48 h. The levels of IFN‐γ, IL‐4 and IL‐10 were measured using an enzyme‐linked immunosorbent assay kit (DuoSet ELISA Development kit, R&D, Minneapolis, MN, USA), according to the manufacturer's protocol.

To examine the suppressive effect of Treg‐of‐B cells, the supernatant from Treg‐of‐B cell–BMDMs in vitro co‐cultures system was collected and TNF‐α and IL‐6 levels were measured. To confirm the anti‐inflammatory effect of Treg‐of‐B cell‐induced M2 macrophages in vivo, lymphocytes isolated from the spleen, ILNs and ALNs of the IMQ mouse were stimulated with anti‐CD3/CD28 antibodies for 72 h. The levels of IL‐22, IL‐17 and IFN‐γ in the supernatant were measured using enzyme‐linked immunosorbent assay.

### 2.12 T cell proliferation

Purified CD4^+^ CD25^−^ (responder) T cells were co‐cultured with different suppressor T cells, including Treg‐of‐B cells and control T cells, in the presence of mitomycin C‐treated splenocytes upon Con A stimulation (2 μg/mL) for 3 days. The suppressive effect was determined by [^3^H] thymidine incorporation, which was added to the cultures for 18 h.

### 2.13 Statistical analysis

Data were analyzed by one‐way analysis of variance, followed by Bonferroni's multiple comparison tests, to calculate between‐group differences, using GraphPad Prism 6.0. Statistical significance was set at *p* < 0.05. *p < 0.05; **p < 0.01; ***p < 0.001.

## 3 RESULTS

### 3.1 Characterization of splenic Foxp3^−^ Treg cells induced by splenic B cells

**FIGURE 1:**
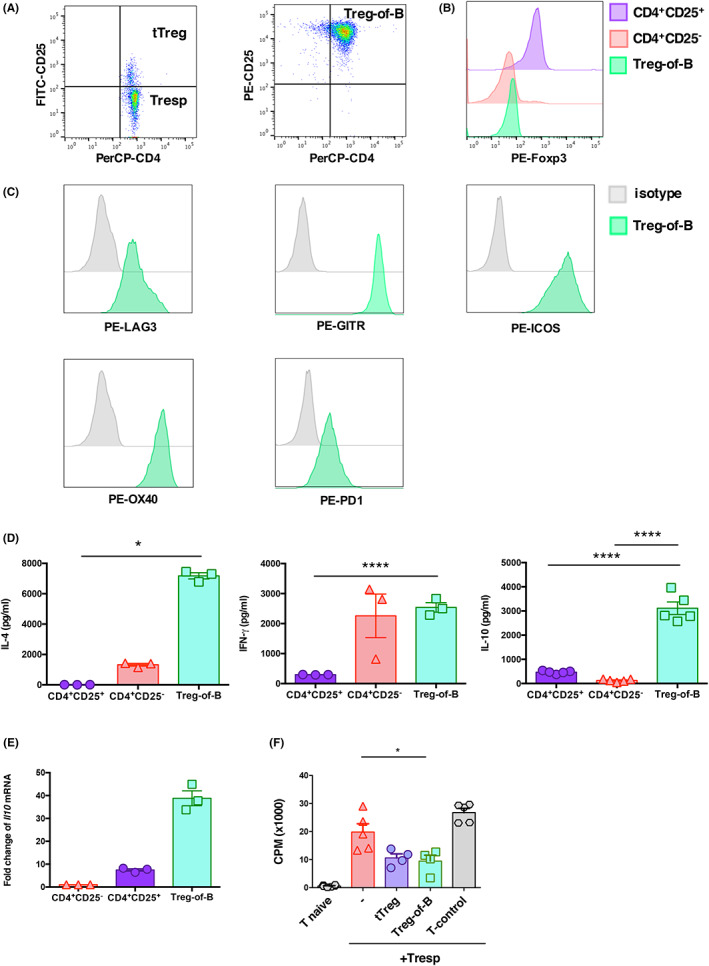
Characteristics of Treg‐of‐B cells. (A) Dot plot represents the expression of CD4 and CD25 in naïve CD4^+^ T cells, tTreg cells and Treg‐of‐B cells. CD4^+^ CD25^+^ cells were isolated as tTreg cells and CD4^+^ CD25^−^ as responder T cells. (B) Expression of Foxp3 in Treg‐of‐B cells compared to CD4^+^ CD25^+^ and CD4^+^ CD25^−^ T cells. (C) Flow cytometry analysis showed the expression of surface markers in Treg‐of‐B cells. (D) Cytokine production by Treg‐of‐B cells, CD4^+^ CD25^+^ and CD4^+^ CD25^−^ T cells were measured by ELISA. (E) We measured the expression of *Il10* mRNA in CD4^+^ CD25^−^ T, CD4^+^ CD25^+^ and Treg‐of‐B cells. The mRNA levels were normalized by housekeeping gene *Gapdh*. (F) Suppressive effect of Treg‐of‐B cells was determined by examining T cell proliferation. The data are representative of two or three independent experiments. The values are expressed as mean ± SEM *p < 0.05, **p < 0.01 and ****p < 0.0001, by one‐way analysis of variance (anova) with Bonferroni's multiple comparison test.

To culture splenic Treg cells induced by splenic B cells, we examined the expression of Treg‐associated markers. The results showed that Treg‐of‐B cells expressed CD25, LAG3, GITR, ICOS, OX40, PD1 and negligibly expressed Foxp3 (Figure 1A–C).

The gating strategy can be found in (Appendix S1: Figure S1). Treg‐of‐B cells produced higher levels of IL‐4, IFN‐γ and IL‐10 than tTreg cells (Figure 1D). We have further confirmed *Il10* mRNA expression using qPCR, which revealed that Treg‐of‐B cells expressed higher levels of *Il10* mRNA than those of tTregs or CD4^+^CD25^−^T cell subsets (Figure 1E).

To examine the suppressive effect of Treg‐of‐B cells, we performed a suppression assay. Although Foxp3 expression was absent, Treg‐of‐B significantly inhibited T cell proliferation (Figure 1F). Therefore, splenic B cells effectively induce Foxp3^−^ Treg cells.

### 3.2 Treg‐of‐B cells induce M2 macrophage polarization

**FIGURE 2:**
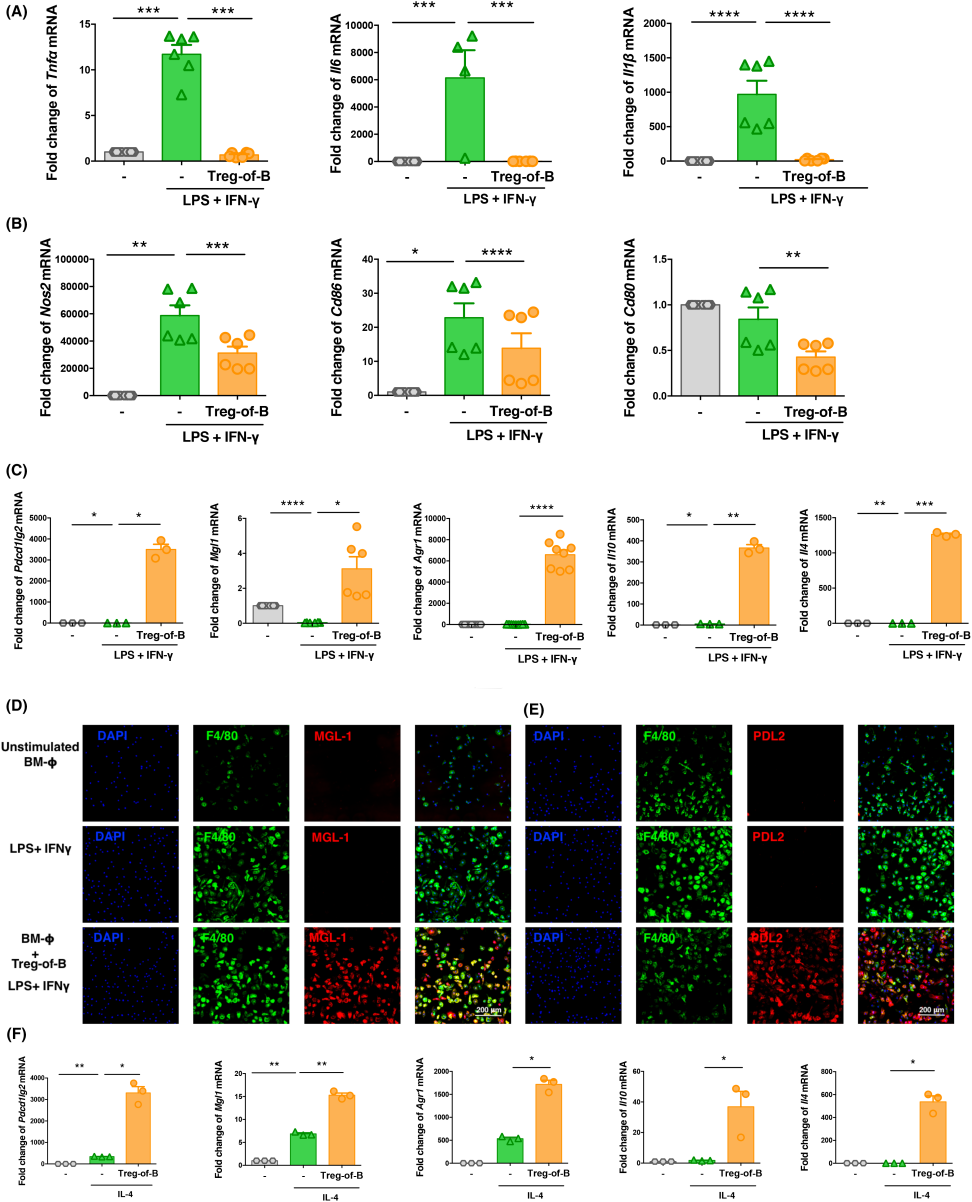
Treg‐of‐B cell‐induced M2 macrophage polarization. (A–C) BMDMs were co‐cultured with Treg‐of‐B cells at 1:3 ratio under anti‐CD3/CD28 stimulation overnight. Subsequently, BMDMs were treated with LPS/IFN‐γ for 24 h. Gene expression levels in BMDMs were determined by RT‐PCR. The mRNA levels were normalized by housekeeping gene *Gapdh*. (D–E) After co‐culturing with Treg‐of‐B cells, the macrophages were subjected to immunofluorescence staining with DAPI (blue), Alexa 647‐F4/80 (green), PE‐PDL2 (red) and PE‐MGL‐1 (red) to visualize their expression (scale bar, 200 μm). (F) BMDMs were cultured together with Treg‐of‐B cells under anti‐CD3/CD28 activation. Next, the cultures were stimulated with IL‐4 for 24 h. The mRNA expression in BMDMs was analyzed by Q‐PCR. ‘‐’(control) group was BMDMs. The data are representative of two or three independent experiments. The values are expressed as mean ± SEM *p < 0.05, **p < 0.01 and ***p < 0.001, ****p < 0.0001, by one‐way analysis of variance (anova) with Bonferroni's multiple comparison test.

We investigated whether Treg‐of‐B could regulate macrophage activation and their phenotype polarization. BMDMs were co‐cultured with Treg‐of‐B cells overnight and stimulated with LPS/IFN‐γ for 24 h. The results showed that the inflammatory cytokine gene transcription of *Tnfα*, *Il6* and *Il1β* in macrophages was suppressed by Treg‐of‐B cells (Figure 2A). Additionally, Treg‐of‐B cells also suppressed the transcriptional expression of macrophage activation markers, such as *Nos2*, *Cd86* and *Cd80* (Figure 2B). These results indicate that Treg‐of‐B can induce tolerance by inhibiting macrophage activation.

Next, we examined whether Treg‐of‐B cells can affect macrophage polarization in vitro. We found that M2‐associated genes, such as *Pdcd1lg2*, *Mgl1*, *Arg1*, *Il10* and *Il4*, were significantly upregulated in macrophages co‐cultured with Treg‐of‐B cells compared to BMDMs cultured alone, even under M1‐polarized conditions (Figure 2C).

Moreover, we examined the protein level of M2 macrophages markers, such as MGL‐1 and PDL2 by immunofluorescence staining. The macrophages were stained with DAPI, Alexa 647‐F4/80, PE‐MGL‐1 and PE‐PDL2. The results revealed that the macrophages co‐cultured with Treg‐of‐B cells co‐expressed F4/80 and MGL‐1 or F4/80 and PDL2, as noted from the merged images (Figure 2D,E).

Moreover, in response to M2‐polarized conditions, Treg‐of‐B cells enhanced the expression of *Pdcd1lg2*, *Mgl1*, *Arg1*, *Il10* and *Il4* in macrophages compared to IL‐4‐treated BMDMs (Figure 2F). These results potentially provided new insights into the anti‐inflammatory effects of Treg‐of‐B cells via the induction of M2 macrophage polarization.

### 3.3 Treg‐of‐B cell‐induced M2 macrophage polarization in a cell contact‐dependent manner

**FIGURE 3:**
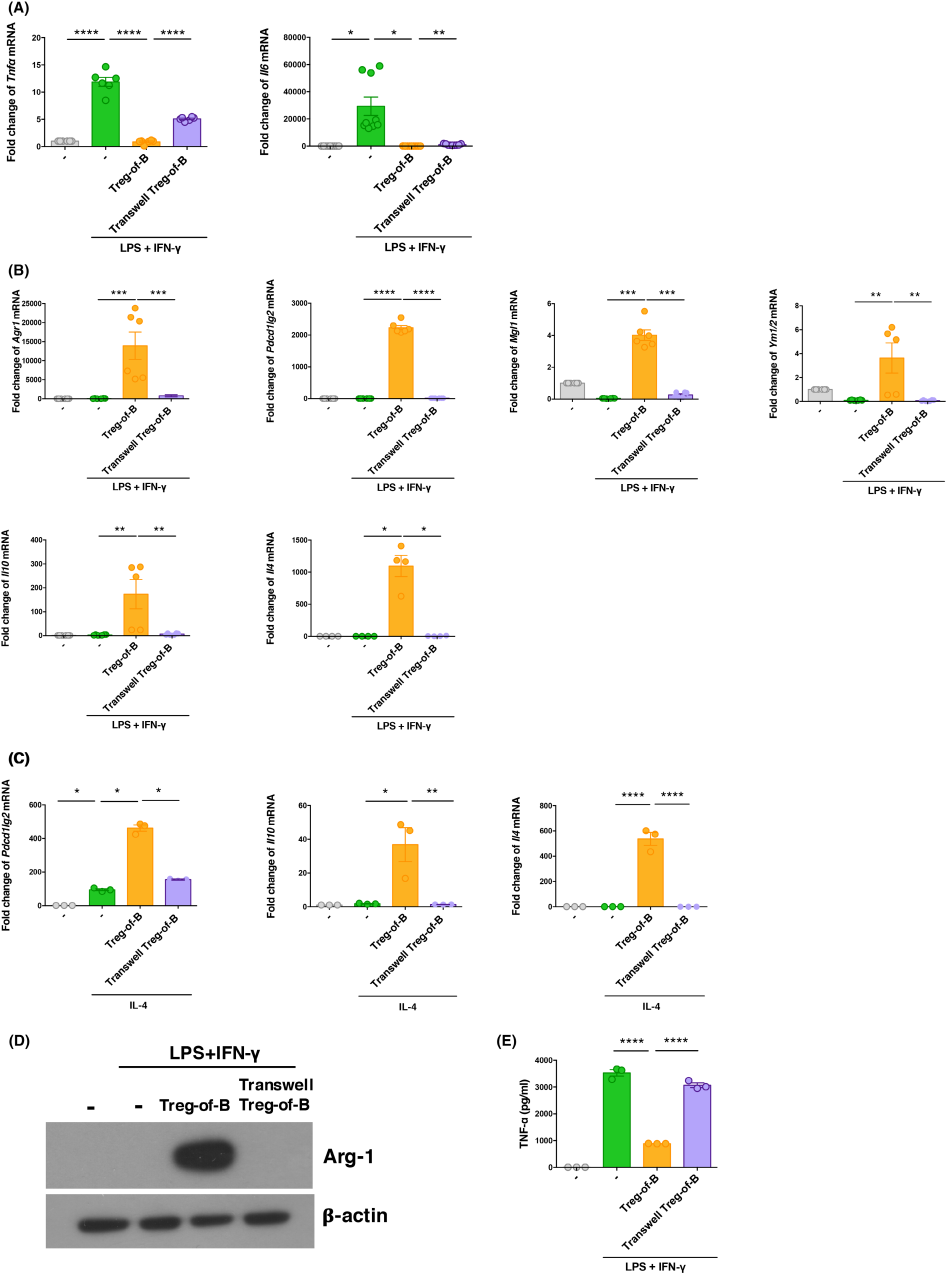
Treg‐of‐B cells promote M2 polarization in a cell contact‐dependent manner. (A–C) BMDMs were co‐cultured with Treg‐of‐B cells at a 1:3 ratio under anti‐CD3/CD28 stimulation overnight in the presence or absence of a Transwell system. The mRNA expression in BMDMs was analyzed after LPS/IFN‐γ or IL‐4 stimulation. (D) The production of Arg‐1 protein in BMDMs was detected by western blotting. (E) TNF‐α production by BMDMs co‐cultured with Treg‐of‐B cells in a Transwell system was measured by ELISA. ‘‐’(control) group was BMDMs. The data are representative of two or three independent experiments. The values are expressed as mean ± SEM *p < 0.05, **p < 0.01 and ***p < 0.001, ****p < 0.0001, by one‐way analysis of variance (anova) with Bonferroni's multiple comparison test.

To further investigate the molecular mechanism of M2 macrophage induction by Treg‐of‐B cells, we co‐cultured Treg‐of‐B cells and BMDMs in a Transwell system. Inhibiting contact between Treg‐of‐B cells and BMDMs significantly reversed the downregulation of *Tnfα* and *Il6* mRNA expression in macrophages compared to that in the co‐culture system (Figure 3A). Moreover, the mRNA expression of the M2 markers, *Arg1*, *Pdcd1lg2*, *Mgl1*, *Ym1/2*, *Il10 and Il4*, was significantly reduced in BMDMs in the Transwell system (Figure 3B). We have measured the M2 markers under IL‐4‐stimulated conditions in transwell systems. Our results showed that the mRNA expression of M2 markers, *Pdcd1lg2*, *Il10* and *Il4* was significantly reduced in BMDMs in the transwell system. These results suggested that Treg‐of‐B cells induced M2 macrophages with cell–cell contact, under IL‐4‐stimulated conditions (Figure 3C).

Western blotting showed that BMDMs co‐cultured with Treg‐of‐B cells produced high levels of Arg‐1 protein (Figure 3D). In contrast, the production of Arg‐1 in BMDMs in the Transwell system disappeared. Moreover, inhibition of the contact between Treg‐of‐B cells and BMDMs significantly reversed TNF‐α production compared to co‐cultures (Figure 3E). Hence, Treg‐of‐B cell‐mediated modulation of macrophage function, including its induction of M2 macrophage polarization, requires cell‐to‐cell contact.

### 3.4 Treg‐of‐B cells induced tolerogenic M2‐like macrophage programming by activating STAT6

**FIGURE 4:**
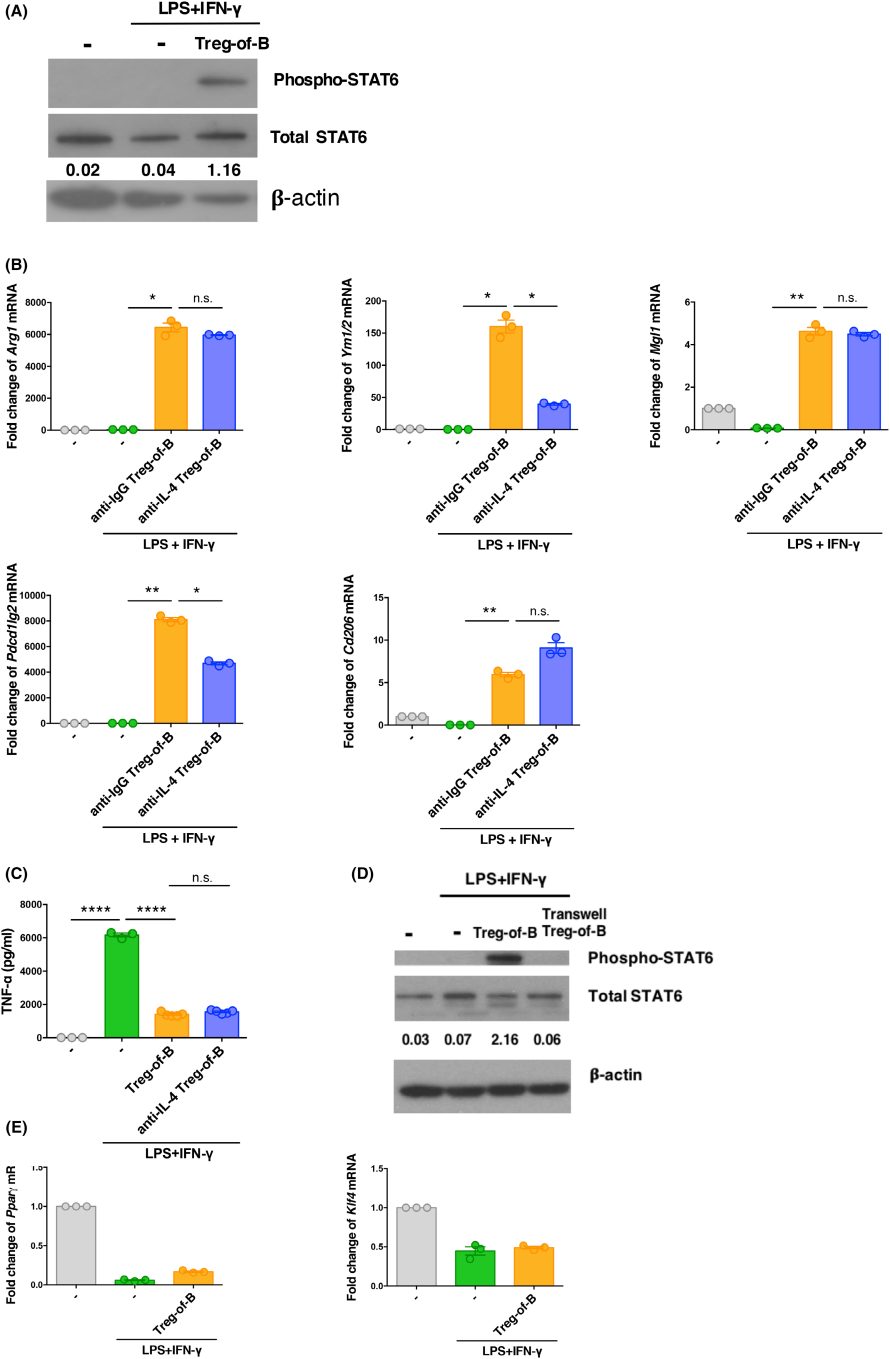
Treg‐of‐B cells induced programming of M2 macrophages via STAT6 activation. (A) Phosphorylated STAT6 and total STAT6 in LPS/IFN‐γ‐stimulated BMDMs co‐cultured with anti‐CD3/CD28 stimulated Treg‐of‐B cells were detected by western blotting. The ratio of phosphorylated STAT6 to the total basal STAT6 protein was quantified by performing densitometry on the immunoblots and analyzed using Image J. (B) IL‐4 neutralizing antibodies were added to the Treg‐of‐B cell‐BMDMs co‐culture system. The mRNA expression in BMDMs was analyzed by Q‐PCR. (C) Inflammatory cytokine production was measured by ELISA. (D) Phosphorylated STAT6 and total STAT6 were detected in BMDMs co‐cultured with Treg‐of‐B cells in the presence or absence of a Transwell system. The ratio of phosphorylated STAT6 to the total basal STAT6 protein was quantified by performing densitometry on the immunoblots and analyzed using Image J. (E) The expression of downstream molecules of the STAT6 pathway in BMDMs co‐cultured with Treg‐of‐B cells was analyzed. The data are representative of two or three independent experiments. ‘‐’(control) group was BMDMs. The values are expressed as mean ± SEM *p < 0.05, **p < 0.01 and ****p < 0.0001; n.s. = not significant. The data were analyzed by one‐way analysis of variance (anova) with Bonferroni's multiple comparison test.

A previous study showed that STAT6 activation promotes M2 macrophage polarization.^32^ Therefore, we investigated whether Treg‐of‐B cells could induce the phosphorylation of STAT6 to induce M2 macrophage polarization. When BMDMs were co‐cultured with Treg‐of‐B cells in the presence of LPS/IFN‐γ stimulation, phosphorylation of STAT6 in BMDMs was observed (Figure 4A).

IL‐4 was critical for the activation of STAT6. Therefore, we hypothesized that IL‐4 plays a role in Treg‐of‐B cell‐induced M2 macrophage polarization. After neutralizing the effects of IL‐4, some, but not all, of the M2‐associated markers were affected by IL‐4 (Figure 4B). We also found that blocking IL‐4 did not affect the suppression of TNF‐α production by Treg‐of‐B cells (Figure 4C). Next, we found that disruption of cell contact between BMDMs and Treg‐of‐B cells downregulated the phosphorylation of STAT6 in BMDMs (Figure 4D). These results indicated that Treg‐of‐B cells promote M2 macrophage polarization via STAT6 activation, rather than via IL‐4, in a cell contact‐dependent manner.

Krüppel‐like factor 4 (KLF4) and peroxisome proliferator‐activated receptor gamma (PPAR‐γ) are downstream molecules of the STAT6 pathway. Thus, we investigated whether these molecules are involved in STAT6 activation by Treg‐of‐B cells. We found that *Pparγ* and *Klf4* were not expressed in BMDMs co‐cultured with Treg‐of‐B cells (Figure 4E). Therefore, *Pparγ* and *klf4* appear not to be downstream molecules of the STAT6 pathway activated by Treg‐of‐B cells.

### 3.5 STAT6 in BMDMs was critical for the induction of M2 macrophages by Treg‐of‐B cells, but no effect on macrophage activation

**FIGURE 5:**
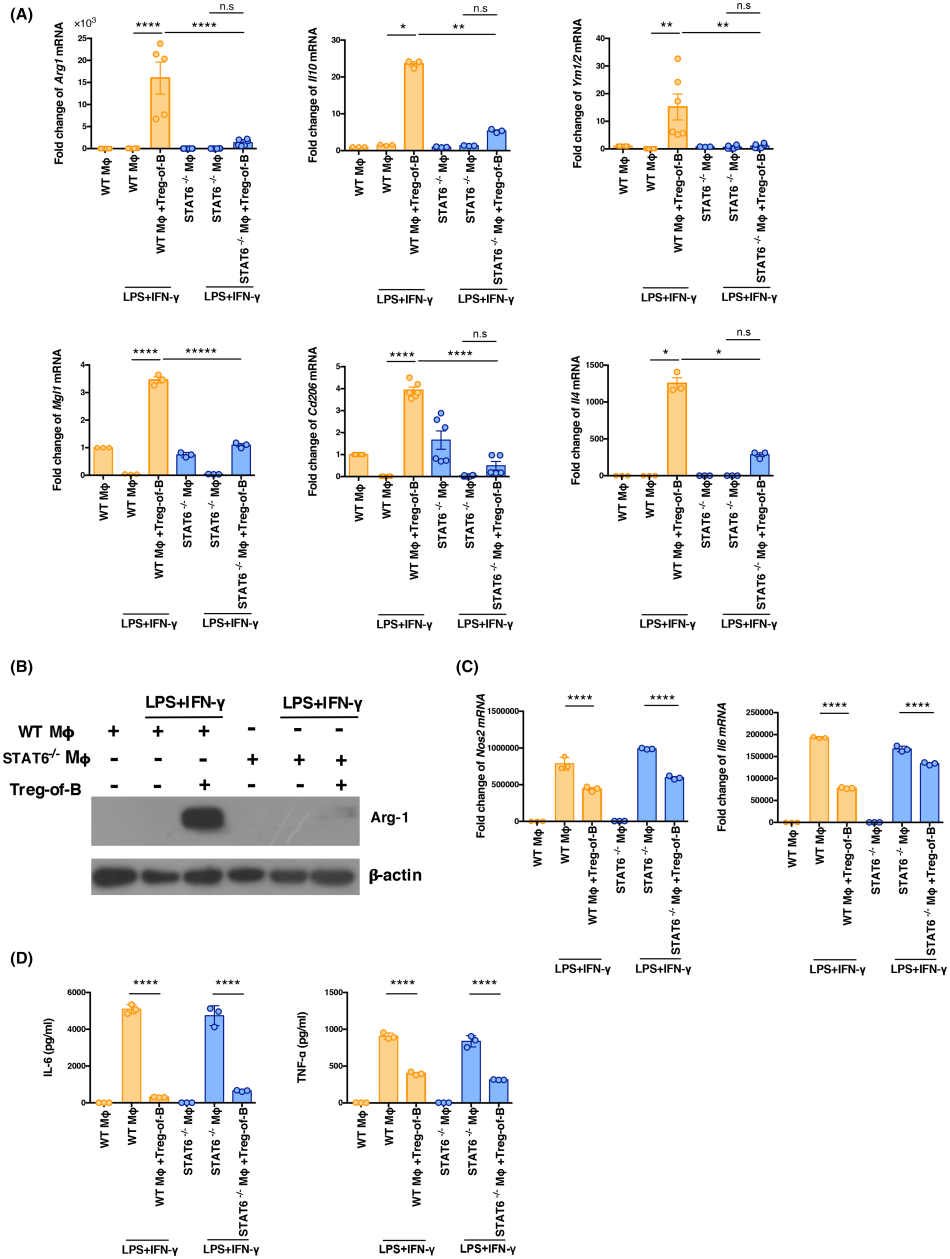
STAT6 played a role in M2 polarization by Treg‐of‐B cells, not in suppressing macrophage activation. (A, C) Wild‐type or STAT6^−/−^ BMDMs were co‐cultured with Treg‐of‐B cells at a 1:3 ratio under anti‐CD3/CD28 stimulation overnight. Next, BMDMs were treated with LPS/IFN‐γ for 24 h. The mRNA expression in BMDMs was analyzed by Q‐PCR. (B) The protein level of Arg‐1 in BMDMs was detected by western blotting. (D) Cytokine production by Treg‐of‐B cell–BMDM co‐cultures were measured by ELISA. The data are representative of two or three independent experiments. The values are expressed as mean ± SEM *p < 0.05, **p < 0.01 and ****p < 0.0001; n.s. = not significant. The data were analyzed by one‐way analysis of variance (anova) with Bonferroni's multiple comparison test.

To confirm the role of STAT6 activation for M2 macrophage polarization by Treg‐of‐B cells, we examined the expression of M2‐associated markers in the absence of STAT6 signalling in BMDMs from STAT6^−/−^ mice. Figure 5A shows that the STAT6^−/−^ BMDM‐Treg‐of‐B cell co‐culture group had significantly reduced *Arg1*, *Il10*, *Ym1/2*, *Mgl1*, *Cd206* and *Il4* gene expression compared to the WT BMDM–Treg‐of‐B cell co‐culture group. We also observed that the M2‐related Arg‐1 protein was markedly reduced in STAT6^−/−^ BMDMs co‐cultured with Treg‐of‐B cells (Figure 5B). In addition, STAT6^−/−^ BMDM co‐cultures suppressed the expression of inflammatory genes, such as *Nos2* and *Il6*, as well as the production of inflammatory cytokines, IL‐6 and TNF‐α (Figure 5C,D). These results indicated that STAT6 activation is critical for inducing alternative macrophage polarization by Treg‐of‐B cells, not affecting macrophage activation.

### 3.6 Treg‐of‐B‐induced M2 macrophages ameliorate skin inflammation in a mouse model of psoriasis

**FIGURE 6:**
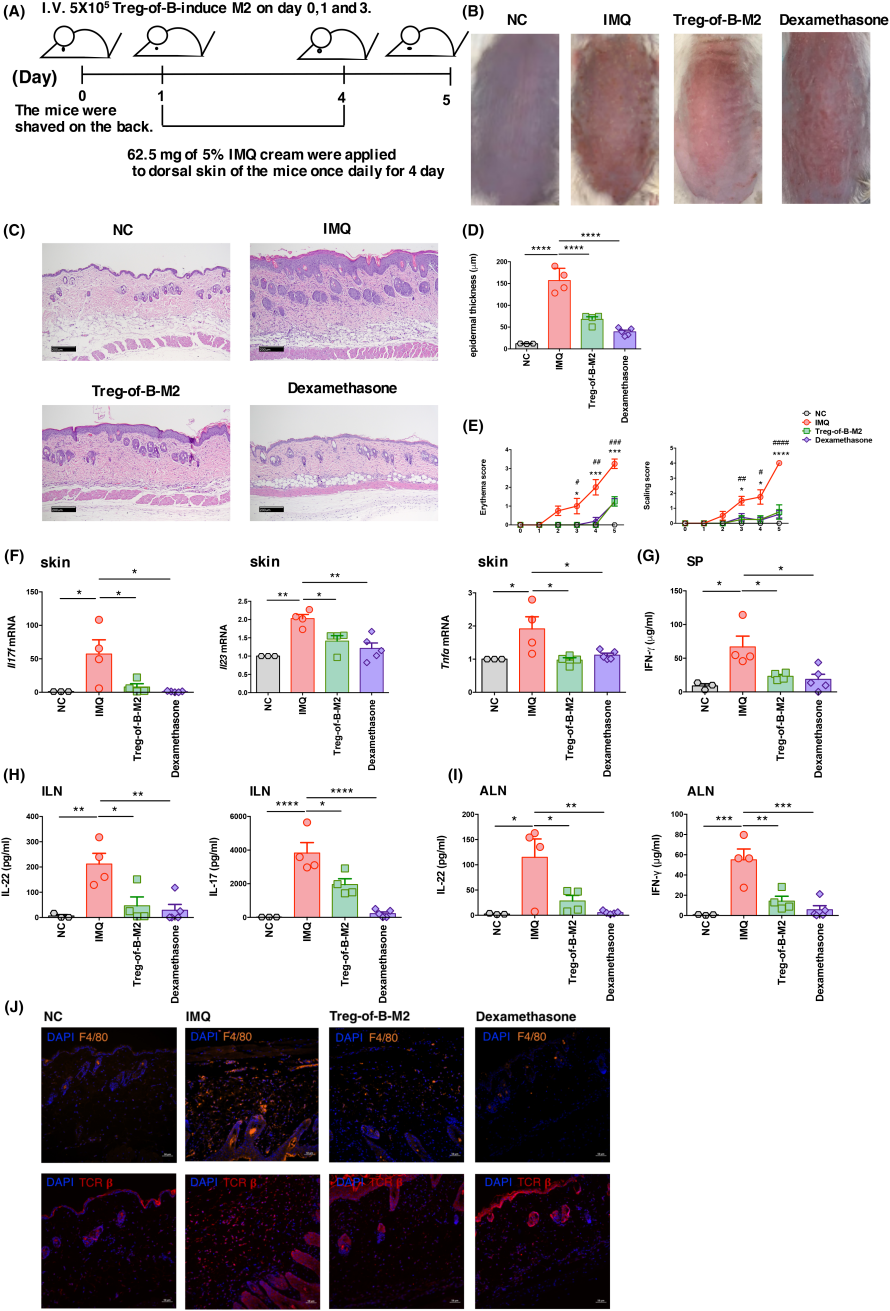
Treg‐of‐B cell‐induced M2 macrophages alleviate psoriasis. (A) IMQ cream was applied to the shaved back skin of mice for 4 consecutive days. Treg‐of‐B cell‐induced M2 macrophages were adoptively transferred to mice via intravenous injection on days 0, 1 and 3. (B) Phenotypical presentation of the back skin in the negative control, IMQ, Treg‐of‐B cell‐induced M2 and dexamethasone groups. (*n* = 3/NC group, *n* = 4/IMQ group, *n* = 4/Treg‐of‐B cell‐induced M2 group, *n* = 5/ dexamethasone groups). (C) The skin sections were stained with haematoxylin and eosin staining to examine the infiltration of immune cells and the thickness of epidermal tissue. (D) To quantify epidermal thickness in the skin section pictures with H and E staining. (E) Scaling and erythema were scored daily. The data are expressed as mean ± SEM *p < 0.05, ***p < 0.001 and ****p < 0.0001 vs Treg‐of‐B‐M2 group. ^#^ *p* < 0.05, ^##^ *p* < 0.01, ^###^ *p* < 0.001 and ^####^ *p* < 0.0001 vs. Dexamethasone group. The data were analyzed by one‐way analysis of variance (anova) with Bonferroni's multiple comparison test. (F) Expression of inflammatory genes in skin tissue was analyzed by Q‐PCR. (G–I) Inflammatory cytokine production was measured in anti‐CD3/CD28 activated cells derived from the spleen, ILNs and ALNs. The data are expressed as mean ± SEM *p < 0.05, **p < 0.001, ***p < 0.001 and ****p < 0.0001, by one‐way analysis of variance (anova) with Bonferroni's multiple comparison test. (J) The skin sections from the psoriatic mouse model were stained with DAPI, Alexa 647‐F4/80 or Alexa 594‐TCR β to examine macrophage (orange) and T cell (red) infiltration in skin tissues (scale bar, 50 μm).

In vitro, we found that Treg‐of‐B cells drove macrophages toward an anti‐inflammatory M2 phenotype. Next, we examined whether Treg‐of‐B‐induced M2 macrophages could be used as immunotherapy for skin inflammation. Mice were treated with 5% IMQ cream on their back for 4 consecutive days, which mimics human psoriasis. The dorsal skin displayed typical features of psoriasis‐like dermatitis, including scaling, erythema and thickening, beginning 2–3 days after IMQ application, with the greatest severity on day 5 (Figure 6A,B). Compared to the IMQ group, skin inflammation, such as erythema and scaling, was significantly attenuated in the Treg‐of‐B‐induced M2‐treated group (Figure 6B).

Histological analysis showed that epidermal thickening and immune cell infiltration were significantly increased in IMQ‐treated mice compared with controls (Figure 6C). In contrast, the Treg‐of‐B‐induced M2‐treated group had decreased epidermal thickness and reduced inflammatory cell infiltration in the dorsal skin. We measured the epidermal thickness in the skin section pictures. The results showed that the treatment of Treg‐of‐B‐M2 macrophages in the psoriasis mouse model significantly decreased the epidermal thickness compared to that of psoriatic mouse group. In addition, the dexamethasone treatment group also showed a significant decrease in epidermal thickness compared to the psoriatic mouse group (Figure 6D). Moreover, Treg‐of‐B‐induced M2 mice had reduced erythema and scaling scores compared to the IMQ‐group (Figure 6E). *Il17f* mRNA, *Il23* mRNA and *Tnfα* mRNA expression in skin lesions was significantly increased in IMQ‐treated mice, and was significantly decreased by the adoptive transfer of Treg‐of‐B‐induced M2 macrophages (Figure 6F).

We further examined T cell activation in the spleen and lymph nodes by measuring cytokine production by anti‐CD3/CD28‐stimulated immune cells isolated from these organs. IL‐22 production by immune cells in the ILNs and ALNs increased in IMQ‐treated mice and was significantly reduced in the Treg‐of‐B‐induced M2 and dexamethasone groups. IFN‐γ production by splenic cells and ALN also showed a similar pattern (Figure 6G–I). In addition, IL‐17 production by T cells in the ILN was significantly decreased in the Treg‐of‐B‐induced M2 group compared to IMQ group (Figure 6H) Therefore, Treg‐of‐B‐induced M2 macrophages decreased the Th1/Th17 response in a mouse model of psoriasis.

We examined the infiltration of T cell and macrophages by immunofluorescence staining. The skin sections were stained with DAPI, Alexa 647‐F4/80 or Alexa 594‐TCR β to examine macrophage (orange) and T cell (red) infiltration in skin tissues. The results showed that there was an increased infiltration of macrophages and T cells in the inflammatory skin tissue of the psoriatic mouse group. In addition, after treatment with Treg‐of‐B cells and dexamethasone, the infiltration of macrophages and T cells decreased compared to that of a control group (Figure 6J). These findings suggest that Treg‐of‐B cell‐induced M2 macrophages alleviate psoriatic skin inflammation by reducing inflammatory cell infiltration in skin lesions and systemic inflammation. Therefore, we demonstrated that Treg‐of‐B‐induced M2 macrophages could ameliorate an M1‐mediated autoimmune disorder.

**FIGURE 7:**
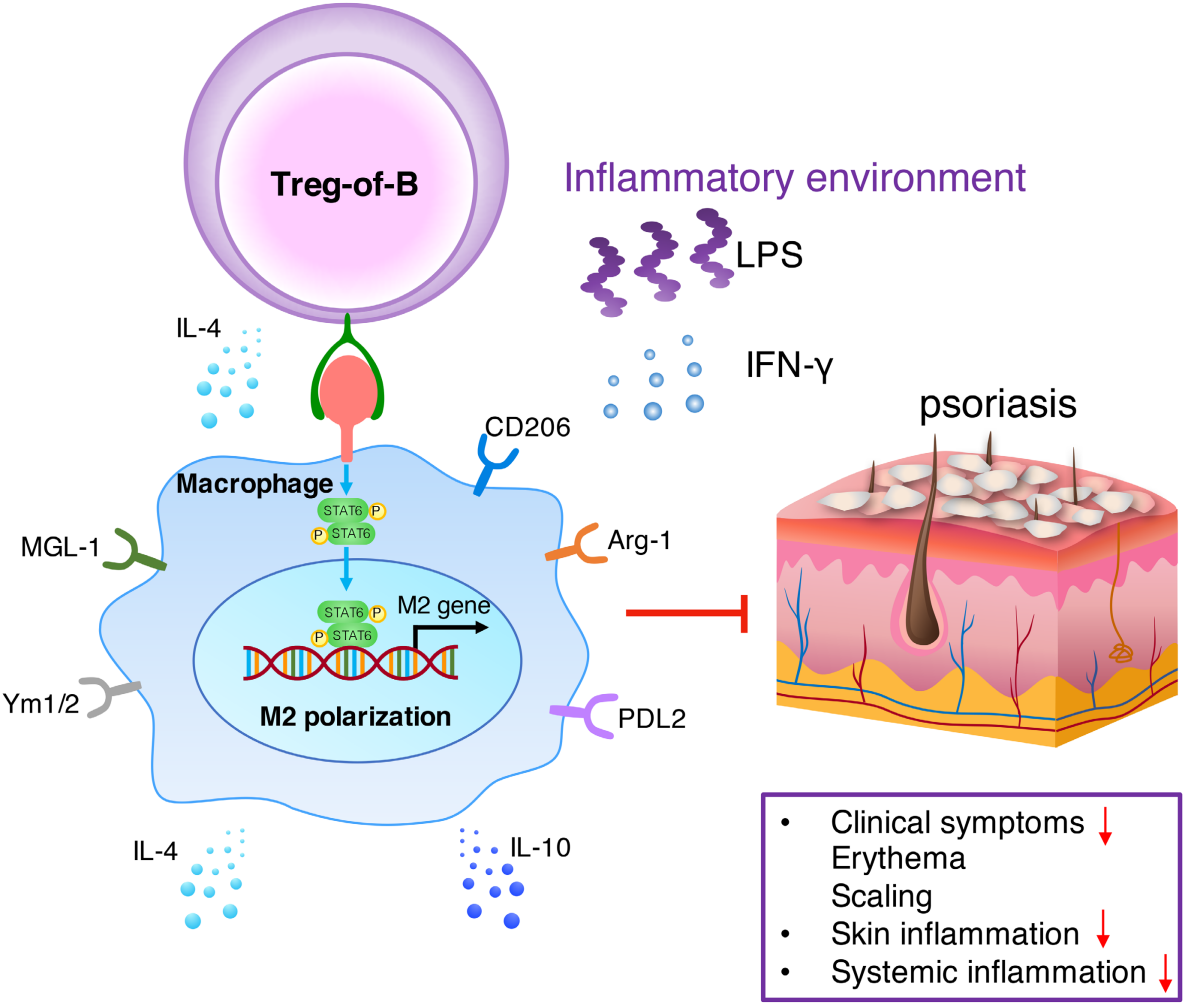
Treg‐of‐B cells alleviate psoriasis by inducing M2 macrophages. Treg‐of‐B cells induce M2 macrophage polarization via STAT6 activation in a cell contact‐dependent manner. Adoptive transfer of Treg‐of‐B cell‐induced M2 macrophages reduces skin and systemic inflammation, such as scaling and erythema in an animal model of psoriasis.

The findings of this study, including the induction of M2 macrophage polarization by Treg‐of‐B cells through STAT6 activation and the therapeutic potential of Treg‐of‐B cells for psoriasis, are summarized in Figure 7.

## 4 DISCUSSION

Appropriate immune‐mediated inflammation can protect the host from microbial infection and mediate tissue regeneration and repair. However, excessive inflammation can lead to the development of many inflammatory disorders. An imbalance in M1 and M2 macrophage polarization has been found to be involved in various diseases. Therefore, understanding the mechanisms involved in maintaining immune homeostasis and tolerance is important. Previous studies indicated that B cells played a role in promoting immune tolerance.^9^, ^33^ Evidence suggests that resting B cells expand and maintain Treg populations in the presence of TGF‐β or allogeneic response.^10^, ^11^, ^34^ Previously, our group reported that B cells derived from the spleen, peritoneal cavity and Peyer's patches have the capacity to induce CD4^+^ CD25^+^ FoxP3^−^ Treg cells in vitro; thus, we termed them ‘Treg‐of‐B cells’.^35^, ^36^ Treg‐of‐B cells protect mice from Th2‐mediated allergic inflammation,^14^ modulate the Th1 response and alleviate collagen‐induced arthritis.^17^ Furthermore, Treg‐of‐B cells suppress Th1 and Th17 responses during intestinal inflammation.^18^ Therefore, these Treg‐of‐B cells can alleviate T cell‐mediated inflammation. In addition, we previously demonstrated that Treg‐of‐B cells inhibited the NLRP3 inflammasome in macrophages via NF‐κB signalling.^19^ In this study, we found that Treg‐of‐B cells promoted M2 polarization via STAT6 activation in a cell‐contact‐dependent manner. In contrast, several studies have reported that the in vitro induction of M2 macrophages is stimulated by canonical soluble factor; M2a macrophages are stimulated by IL‐4/IL‐13 and M2c macrophages are stimulated by IL‐10.^37^, ^38^, ^39^ In addition, a previous study indicated that human CD4^+^ CD25^+^ FoxP3^+^ Treg cells induce alternative CD163^+^/CD206^+^ human monocytes/macrophages in both a cell contact‐ and soluble factor‐dependent manner.^27^ It was suggested that Treg‐mediated induction of CD206^+^ alternative macrophages occurred in a cytokine‐independent manner, while induction of CD163^+^ expression in human macrophages was partially dependent on IL‐10. In our study, Treg‐of‐B cell‐mediated induction of M2 macrophages mainly occurred in a cell contact‐dependent manner, partially via IL‐4 (Figure 3 and Figure 4). In addition, Treg‐of‐B cell‐mediated suppression of macrophages is dependent on cell contact, and involves decreased TNF‐α production. It has been suggested that Treg‐of‐B cells could modulate innate immunity by inducing M2 macrophages and maintaining immune homeostasis, which may involve a novel cell contact mechanism.

Previous studies indicated that activation of STAT6 by IL‐4/IL‐13 leads to M2 macrophage polarization, which is associated with immune suppression and tissue repair.^32^, ^40^ KLF4, which is downstream of STAT6, is involved in promoting M2 macrophage function by suppressing NF‐κB/hypoxia inducible factor 1α‐dependent transcription. Moreover, STAT6 cooperates with both PPAR‐γ and KLF4, which promotes M2 macrophage function.^41^, ^42^, ^43^ Our results showed that Treg‐of‐B cells induced M2 macrophage polarization by inducing STAT6 phosphorylation (Figure 4 and Figure 5) in a cell contact‐dependent manner. However, IL‐4 did not play a significant role in inducing M2 macrophages via Treg‐of‐B cells. PPAR‐γ and KLF4 were not downstream molecules of the STAT6 signalling pathways that were induced by Treg‐of‐B cells. Further studies are needed to identify the downstream molecules of STAT6 activation by Treg‐of‐B cells.

Macrophages are heterogeneous innate immune cells that exhibit high plasticity. A previous study indicated that the distribution of activated macrophages in psoriatic skin plays a key role in uncontrolled cutaneous inflammation.^44^, ^45^ Increased M1 macrophage polarization is associated with psoriasis severity.^22^ Therefore, the modulation of macrophage polarization may be a novel therapeutic target for psoriasis.

In the pathogenesis of psoriasis, the activated APCs, such as macrophages and dendritic cells lead to Th cells differentiation into IFN‐γ‐producing T cells and IL‐17‐producing Th17 cells.^46^ The dysregulated T cells infiltrated into the dermis of inflamed skin, which contributed to amplifying the immune response and causing the hyperproliferation of keratinocytes.^47^ In addition, the downstream effector molecules of Th1 and Th17 pathways such as IFN‐γ, IL‐17 and IL‐22, also play a role in the development of psoriatic dermatitis.^47^ In our study, the treatment of Treg‐of‐B‐induced M2 macrophages decreased the IL‐17 production in skin lesions, as well as the IFN‐γ, IL‐17 and IL‐22 production by T cells in LNs compared to those of psoriatic mice. Additionally, histological examination with H and E staining revealed decreased epidermal thickness and reduced inflammatory cell infiltration in the dorsal skin after administration of Treg‐of‐B‐induced M2 macrophages. These results suggested that Treg‐of‐B‐induced M2 macrophages would modulate Th1 and Th17 responses and sequence alleviated psoriatic symptoms, contributing to decreased epidermal thickness and scaling and erythema.

Conventional treatments, including corticosteroids, vitamin D3 analogues, cyclosporine, calcineurin inhibitors, methotrexate and phototherapy, have been widely used for the treatment of patients with psoriasis. Biological therapies have also been developed to target the cytokine pathway for treating psoriasis. However, most of these therapeutic agents have adverse side effects, such as hypertension, renal toxicity, nausea, leukopenia and elevated liver transaminase, suggesting a need for the development of alternative treatments with minimal side effects. In our study, we found that administering Treg‐of‐B cell‐induced M2 macrophages significantly improved the symptoms of psoriasis, including reduction of erythema and scaling. The ameliorative effect was comparable to that observed in mice treated with dexamethasone. Moreover, treatment with Treg‐of‐B‐induced M2 macrophages had no significant side effects. Therefore, the results of this study shed light on a possible novel cell‐based therapeutic approach for autoimmune diseases, such as psoriasis.

In conclusion, we demonstrated that Treg‐of‐B cells induced STAT6 phosphorylation in macrophages, which in turn skewed macrophages toward the M2 phenotype in a cell contact‐dependent manner. M2 macrophages were induced by Treg‐of‐B cells to express *Arg1*, *Cd206*, *Mgl1*, *Pdcd1lg2*, *Ym1*/2, *Il10* and *Il4*. The administration of Treg‐of‐B‐induced M2 macrophages alleviated the manifestations of psoriasis, such as erythema and scaling, in mice treated with IMQ. Furthermore, treatment with Treg‐of‐B‐induced M2 macrophages in psoriasis resulted in a reduction in local inflammation, including decreased skin *Il17f*, *Il23* and *Tnfα* expression, immune cell infiltration and skin epidermal thickness. Moreover, Treg‐of‐B‐induced M2 macrophages modulate systemic Th1 and Th17 responses by decreasing IFN‐γ, IL‐17 and IL‐22 production in draining lymph nodes. Our results demonstrated that Treg‐of‐B cells promote M2 macrophage polarization and that these M2‐polarized macrophages might provide a novel approach for the amelioration of psoriasis and other autoimmune disorders.

## AUTHOR CONTRIBUTIONS

**Jing‐Hui Huang:** Conceptualization (lead); data curation (lead); writing – original draft (lead). **Yu‐Li Lin:** Resources (equal). **Li‐Chieh Wang:** Resources (equal). **Bor‐Luen Chiang:** Funding acquisition (lead); writing – review and editing (lead).

## CONFLICT OF INTEREST STATEMENT

The authors have declared no competing interests exist.

## Supporting information


Appendix S1.

## Data Availability

The relevant data in this study are available from the corresponding author upon reasonable request.
