# Supplementary material for: M2‐like macrophages polarized by Foxp3− Treg‐of‐B cells ameliorate imiquimod‐induced psoriasis
Source: J Cell Mol Med. 2023 Apr 19;27(11):1477–92. doi: 10.1111/jcmm.17748 (PMC10243160; doi:10.1111/jcmm.17748)
Supplement: Supplementary file 1 — Appendix S1. [file JCMM-27-1477-s001.pdf]

Supplementary information

M2-like Macrophages Polarized by Foxp3$^-$ Treg-of-B cells Ameliorate Imiquimod-Induced Psoriasis

Jing-Hui Huang[1], Yu-Li Lin[2], Li-Chieh Wang[3] and Bor-Luen Chiang[1,2,3,4*]

[1]Graduate Institute of Clinical Medicine, National Taiwan University, Taipei, Taiwan

[2]Department of Medical Research, National Taiwan University Hospital, Taipei, Taiwan

[3]Department of Pediatrics, National Taiwan University Hospital, Taipei, Taiwan

[4]Graduate Institute of Immunology, College of Medicine, National Taiwan University, Taipei, Taiwan

*Correspondence author at: Department of Pediatrics, National Taiwan University Hospital, No. 1, Changde St., Zhongzheng Dist., Taipei, Taiwan.

Phone: +886-2-2312-3456 ext. 67302; FAX: +886-2-2311-9087; E-mail address: gicmbor@ntu.edu.tw (B.-L. Chiang).

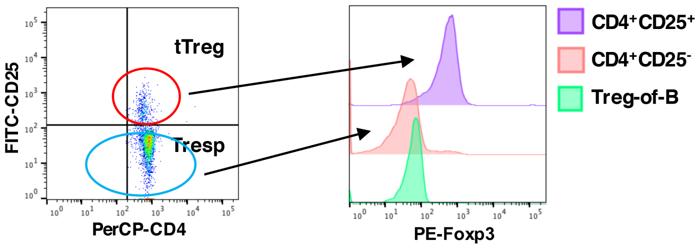

Figure. S1 The gating strategy of Figure 1A and 1B.

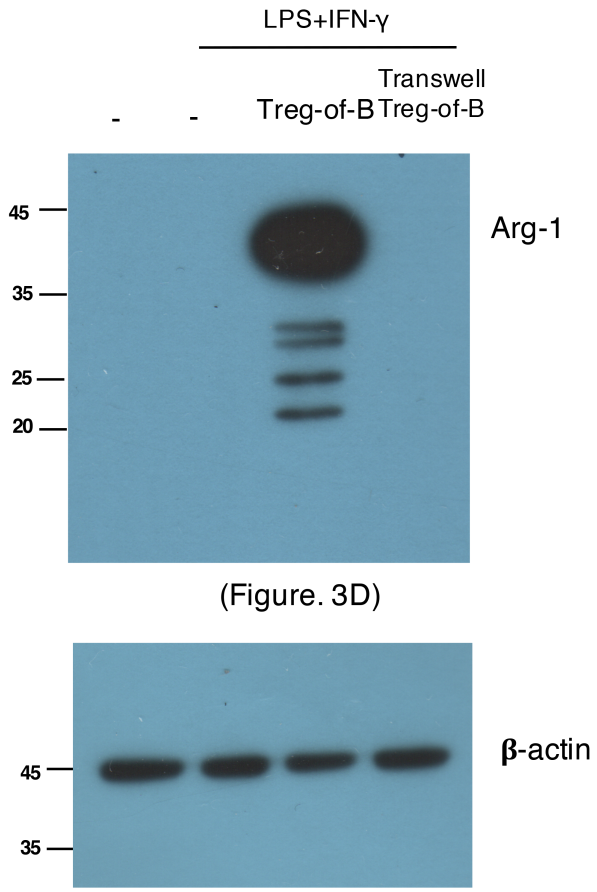

(Figure. 3D)

(Figure. 3D)

Figure. S2. Uncropped image of western blot used to generate Figure. 3D

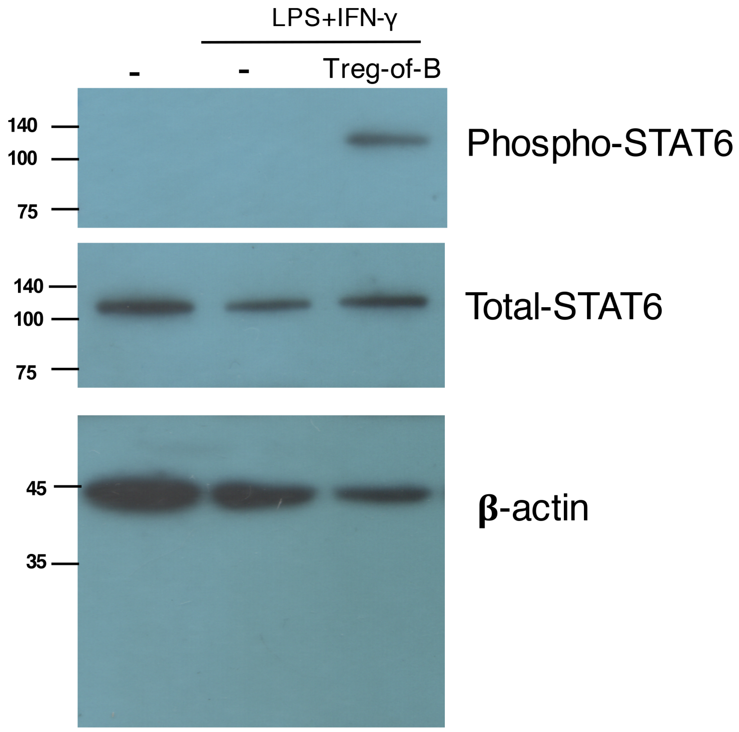

(Figure. 4A)

Figure. S3. Uncropped image of western blot used to generate Figure. 4A

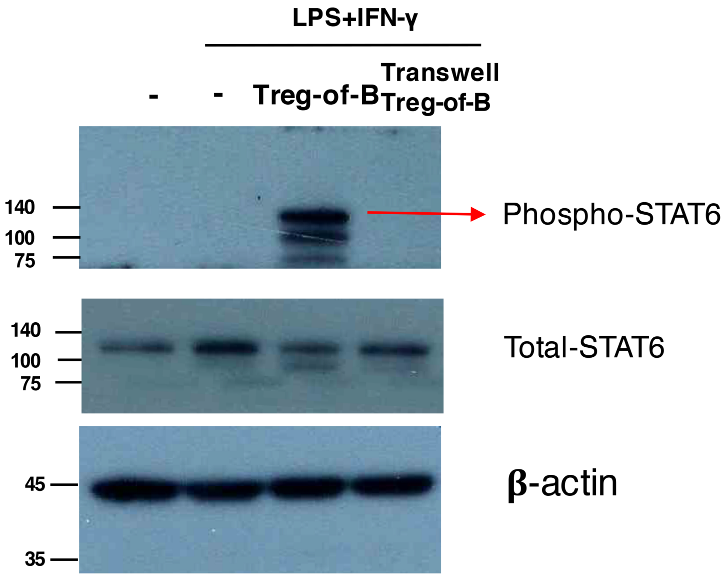

(Figure. 4D)

Figure. S4. Uncropped image of western blot used to generate Figure. 4D

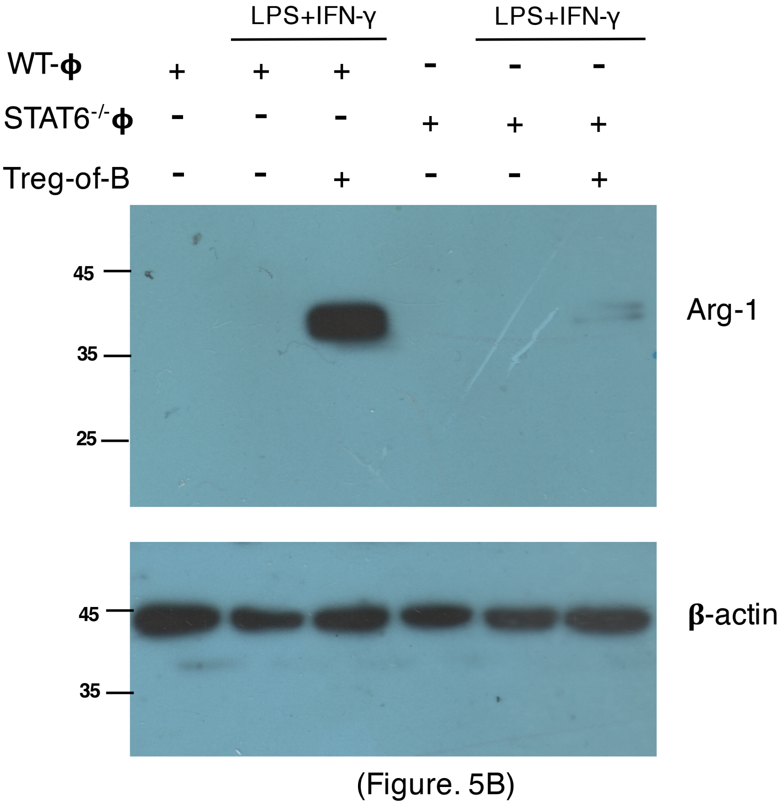

Figure. S5. Uncropped image of western blot used to generate Figure. 5B.
